# Characterization of Reconstructed Human Epidermis in a Chemically-Defined, Animal Origin-Free Cell Culture

**DOI:** 10.1016/j.xjidi.2024.100298

**Published:** 2024-06-28

**Authors:** Julia Bajsert, Valérie De Glas, Emilie Faway, Catherine Lambert de Rouvroit, Miguel Pérez-Aso, Paul W. Cook, Yves Poumay

**Affiliations:** 1 NARILIS-Faculty of Medicine, University of Namur, Namur, Belgium; 2 Provital S.A., Barcelona, Spain; 3 AvantBio Inc, Vancouver, Washington, USA

**Keywords:** Animal-free culture, Keratinocytes, Reconstructed human epidermis, Tissue engineering

## Abstract

The Reconstructed Human Epidermis (RHE) model derived from epidermal keratinocytes offers an ethical and scientific alternative to animal experimentation, particularly in cutaneous toxicology and dermatological research, where the elimination of animal cruelty is of paramount importance. Thus, we compared commercially available chemically defined animal origin-free (cdAOF) supplements, designed for regenerative medicine, to the widely utilized supplement (human keratinocyte growth supplement), which contains growth factors and bovine pituitary extract. Herein we present the extended characterization of RHE derived from newborn, adult, and immortalized N/telomerase reverse transcriptase keratinocytes under cdAOF conditions. Culture of RHE in the cdAOF media produced histological features that were similar to that produced using human keratinocyte growth supplement, with the exception that the basal keratinocytes were less cylindrical. Additionally, immunolocalization of involucrin in the basal layer and increased mRNA expression of several inflammatory-proliferative markers were observed under cdAOF conditions. In RHEs cultured in cdAOF media, expression and immunolocalization of other expected markers of keratinization were similar, while monitoring of barrier function (transepithelial electrical resistance) revealed results that were statistically equal to, or lower than those observed in RHE cultured in human keratinocyte growth supplement. Our study indicates that reconstruction of RHE was accomplished under cdAOF culture conditions and that further refinement could promote an expanded use beyond regenerative medicine, for in vitro toxicology applications.

## Introduction

The disadvantages of using human or non-human animal-originated products (e.g., serum, plasma, platelet lysates, serum-derived albumin, serum-derived transferrin, pituitary extract) or feeder layer cells (eg, immortalized murine 3T3 fibroblasts, human dermal fibroblasts) in cell culture media have been emphasized since the inception of mammalian cell culture. For example, using animal-originated products can increase the expense of culturing mammalian cells, increase inter-lot variability (Baker, 2016; Habanjar et al, 2021), and increase the risk of infection with known human pathogens that include Hepatitis, HIV, or mycoplasma, and transmissible spongiform encephalopathies (eg, bovine spongiform encephalopathy/mad cow disease, chronic wasting disease, Creutzfeldt-Jakob (Hawkes, 2015; Wessman and Levings, 1999). Other known, or even yet-to-be-characterized human and zoonotic infective agents, also pose risks to future cell- and tissue-based therapy (Burnouf et al, 2016). Because of these risks, increased regulatory pressure exists to develop chemically defined animal origin-free (cdAOF) cell culture systems for clinical applications. Collectively, the development and use of cdAOF cell culture supplements and media are thought to promote safer, pathogen-free cell cultures for cells, engineered tissue, and engineered organs for human therapy (Duarte et al, 2023; van der Valk et al, 2018).

Over the last several decades, research and development efforts have been made to reduce or eliminate animal-originated products from cell culture media used for normal non-transformed, non-immortalized cells. As an example, for human neonatal (HEKn) and adult (HEKa) epidermal keratinocyte cell culture, Dulbecco's Modified Eagle Medium/Nutrient Mixture F-12, serum, cholera toxin and murine 3T3 feeder layer cells (Rheinwald and Green, 1977, 1975) were replaced with improved modified low-calcium MCDB 153 basal medium, or low-calcium EpiLife medium, purified growth factors and animal originated products that included bovine pituitary extract (BPE), BSA or bovine serum-derived transferrin (Boyce and Ham, 1983; Peehl and Ham, 1980; Shipley and Pittelkow, 1987; Tsao et al, 1982). Some of these epidermal keratinocyte cell culture systems were characterized as defined, serum-free, or feeder layer-free, but not cdAOF. Eventually, a cdAOF human keratinocyte cell culture system, Supplement S7 (a.k.a. S-70203, S70203) containing growth factors, hormones, and other proteins was reported in 2004 for use with EpiLife basal medium (Cook et al, 2004a). Using the cdAOF Supplement S7 - EpiLife system, it was shown that HEKa could be efficiently isolated in primary culture and serially propagated, with the requirement for pre-coating the cell culture substrate with recombinant human collagen-1. Importantly, HEKa reared in the Supplement S7 - EpiLife system was demonstrated to form a cdAOF reconstructed human epidermis (RHE) that was histologically similar to the human epidermis, but with a less columnar basilar epidermis, a compacted stratum corneum with premature basal cell layer expression of involucrin (IVL). Importantly, this work demonstrated the feasibility of an engineered mammalian tissue (RHE) generated from primary cell culture under complete cdAOF cell/tissue culture conditions.

More recently, an improved cdAOF cell culture supplement system (high keratinocyte serum [HKS]daFREE + human keratinocyte growth supplement [HKGE] in EpiLife basal medium) was developed primarily for cutaneous and corneal regenerative medicine applications (Cook et al, 2012a, 2012b). This cdAOF cell culture system allowed for the rapid and efficient primary isolation and serial propagation of HEKn and HEKa, as well as human corneal epithelial cells. Compared to Supplement S7, post-primary propagation was not dependent on precoating the cell culture surface with recombinant human collagen 1. This improved cdAOF HEKn culture system also supported the generation of RHE that was histologically similar to other RHE models, as well as to normal human skin (Cook et al, 2012a). To demonstrate the potential utility of this improved cdAOF cell culture system in regenerative medicine, HEKn was recently isolated and propagated in EpiLife basal medium plus the HKSdaFREE + HKGE supplements and then utilized as a cdAOF epidermal keratinocyte bioink to generate xenofree, fully vascularized full-thickness 3-Dimensional-bio printed skin equivalents, that were successfully engrafted onto mice (Baltazar et al, 2023). Finally, using a related variant cdAOF supplement system (human fibroblast supplement [HFSdaFREE + HFGE + HFGE2]), efficient primary and post-primary propagation of human dermal fibroblasts as well as corneal fibroblasts were demonstrated as well (Cook et al, 2012a, 2012b). Collectively, cdAOF cell culture supplements represented a breakthrough for the improved cdAOF culture of human skin- and cornea-derived cells, for potential use in cell- or engineered tissue-based therapies.

Since a ban on animal testing of cosmetic products was announced and then rendered efficient in 2013 by the European Union, it prompted the development of alternatives to previous tests that use live animals for skin studies. Additionally, the reduction of animal cruelty by the elimination of animal-originated cell culture media components, such as fetal bovine serum, BPE, transferrin, and BSA from keratinocyte and RHE culture media, has also been of interest. Within this context, models reconstructing human skin by cell culture were developed and characterized, before evaluation and acceptance for cosmetic testing were recognized under the current Organization for Economic Co-operation and Development guidelines 431 and 439 (OECD 2021). Protocols were therefore published to widely spread know-how of epidermal reconstruction and the Open-Source principle has been proposed and applied for collective contribution to potential improvements (Groeber et al, 2016; Mewes et al, 2016). Such models are of course now shown to be scientifically valid and practical and are increasingly used in Europe and worldwide (Coquette et al, 2003; do Nascimento Pedrosa et al, 2017; Poumay et al, 2004). Unfortunately, the elimination of all animal-originated (including human-originated) products from the cell culture media used to generate and validate RHE and full-thickness skin models used for the testing of cosmetic ingredients has not been reported yet in the peer-reviewed literature.

To create epidermal substitutes such as RHE, the availability of primary keratinocytes or immortalized keratinocytes capable of keratinization, in addition to appropriate basal media, their growth supplements, and adequate porous membranes for tissue growth at the air-liquid interface are all critical (Rosdy and Clauss, 1990). The COVID-19 pandemic, unfortunately, provoked sporadic interruptions in the supply chain of essential products (Moran et al, 2022), including the human keratinocyte growth supplement (HKGS) required to support epidermal reconstruction when using EpiLife medium. Consequently, many laboratories around the world relying on HKGS were impeded to reconstruct the epidermis. Seeking an alternative to HKGS, we identified cdAOF supplements provided by AvantBio corporation (HKSdaFREE + HKGE and HFSdaFREE) as an interesting option for generating RHE models for evaluation and acceptance under Organization for Economic Co-operation and Development cosmetic testing guideline 431 and 439 (OECD, 2021, 2015). Additionally, cdAOF supplements not only address the expectations of cosmetic testing’s ethical and marketing advantages (eg, being animal cruelty-free) but also the advantages of a safer and diminished transmissible disease risk for reconstructive and regenerative medicine (Baltazar et al, 2023).

In our current study, we compare the standard animal product (BPE)-containing HKGS supplement previously described for RHE production (Dijkhoff et al, 2021; Hall et al, 2022; Lemper et al, 2014) to the cdAOF supplements HKSdaFREE, HKSdaFREE + HKGE and the fibroblast-specific HFSdaFREE (Cook et al, 2012a, 2012b). Additionally, to obtain a broad, cell-specific performance comparison on all the tested supplements, RHEs were produced from primary HEKn, primary HEKa, and immortalized N/telomerase reverse transcriptase (TERT) keratinocytes (Dickson et al, 2000).

## Results

### The cdAOF supplements produce RHEs that display an epidermal microanatomy and functionality that is similar to RHEs produced using animal product-containing supplement (HKGS)

**Figure 1:**
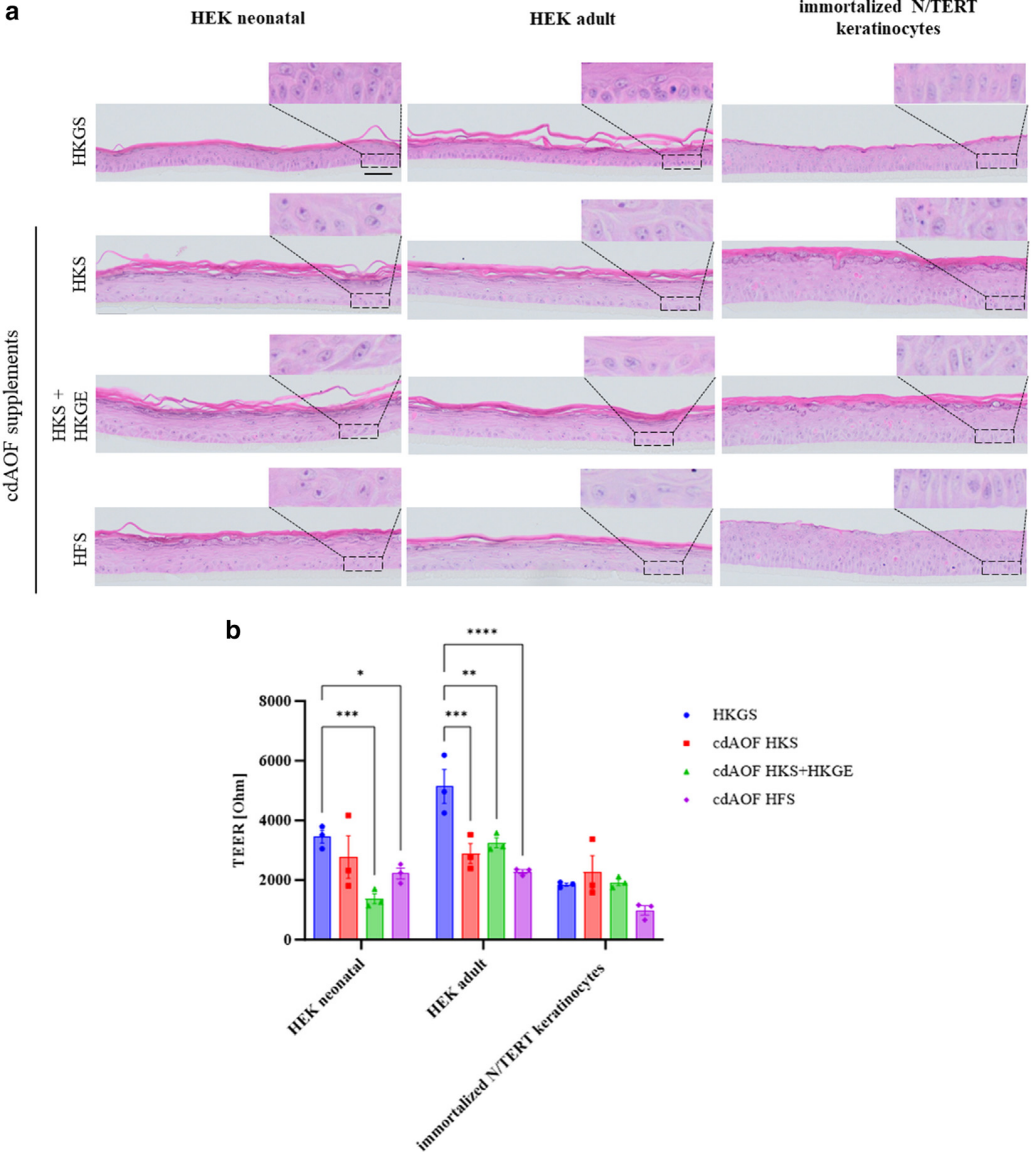
**Microanatomy and barrier differences of the skin models reconstructed with HEK, HEK, or immortalized N/TERT keratinocytes, as cultured with HKGS, HKS, HKS + HKGE, or HFS supplement, respectively.** (**a**) Histological sections of RHE stained with H&E. Scale bar = 50 μm. Pictures are representatives of 3 independent experiments. Magnification (×100). (**b**) TEER measurements performed on RHE (n = 3 independent experiments). Data represent mean ± SEM. Two-way ANOVA was performed, followed by Dunett`s tests. ∗*P* < .05, ∗∗*P* < .01, ∗∗∗*P* < .001, ∗∗∗∗*P* < .0001. HEK, human epidermal keratinocytes; HFS, human fibroblast supplement; HKGE, human keratinocyte growth supplement; HKGS, Human keratinocyte growth supplement; HKS; high keratinocyte serum-free medium; RHE, reconstructed human epidermis; TEER, trans-epidermal electrical resistance; TERT, telomerase reverse transcriptase.

In vitro, 3-Dimensional skin models were reconstructed using HEKn, HEKa, or N/TERT keratinocytes. Each type of RHE was cultured with HKGS, HKS, HKS + HKGE, or HFS supplement as indicated in Figure 1 (also see Table 1). To evaluate the morphology of RHE, H&E staining was performed, revealing that at the face, every RHE corresponded to a fully differentiated and stratified epidermis, that included typical basal and suprabasal layers, a granulated layer, and a keratinized stratum corneum (Figure 1a). However, as compared to RHE cultured in HKGS, some differences were noted, as basal keratinocytes in RHE models derived from all three cell types that were reconstructed using cdAOF media exhibited less frequent cylindrical cell morphology in the basal layer when compared to RHEs produced with HKGS. Interestingly, basal keratinocytes in skin models made of immortalized N/TERT keratinocytes displayed a cylindrical shape when using HFS, which was not the case in HEKn and HEKa.

**Table 1:** Description of Supplements Used for Epidermal Reconstruction

Supplements Analyzed	HKGS	HKS^1^	HKS^1^ + HKGE	HFS^2^
cdAOF^3^	No	Yes	Yes	Yes
Human cells for which the supplement was designed	epidermal keratinocytes	epidermal keratinocytes and corneal epithelial cells	epidermal keratinocytes and corneal epithelial cells	dermal fibroblasts

Abbreviations: cdAOF, chemically-defined animal origin-free; HFS, human fibroblast supplement; HKGS, human keratinocyte growth supplement; HKGE, human keratinocyte growth supplement; HKS, high keratinocyte serum.

1 HKSdaFREE.

2 HFSdaFREE.

3 Chemically-defined animal origin-free.

The barrier function of cdAOF and HKGS-generated RHEs was evaluated by transepithelial electrical resistance (TEER) measurement (Figure 1b). Comparing RHEs derived from HEKn cultured in HKGS versus HKS produced no statistically significant difference in the TEER values (3450 Ohms for HKGS versus 2750 Ohms for HKS). In contrast, comparing RHEs derived from HEKn cultured in HKGS versus HKS + HKGE produced a statistically significant difference (lowering) in the TEER values (3450 Ohms for HKGS versus 1350 Ohms for HKS). Comparing RHEs derived from HEKa cultured in HKGS versus HKS produced statistically significant lowering in the TEER values (5100 Ohms for HKGS versus 2900 Ohms for HKS) and in a similar fashion, comparing RHEs derived from HEKa cultured in HKGS versus HKS + HKGE, produced a statistically significant lowering of the TEER values as well (5100 Ohms for HKGS versus 3250 Ohms for HKS + HKGE). When examining RHEs derived from RHEs generated from either HEKn or HEKa cultured in HFS-supplemented media, resulted in a statistically significant lowering of TEER values. Finally, no significant difference in TEER values was observed between any of the supplements on RHEs derived from immortalized N/TERT keratinocytes.

### Assessment of cellular proliferation via Ki67 expression in RHEs derived from HEKn, HEKa or N/TERT keratinocytes

**Figure 2:**
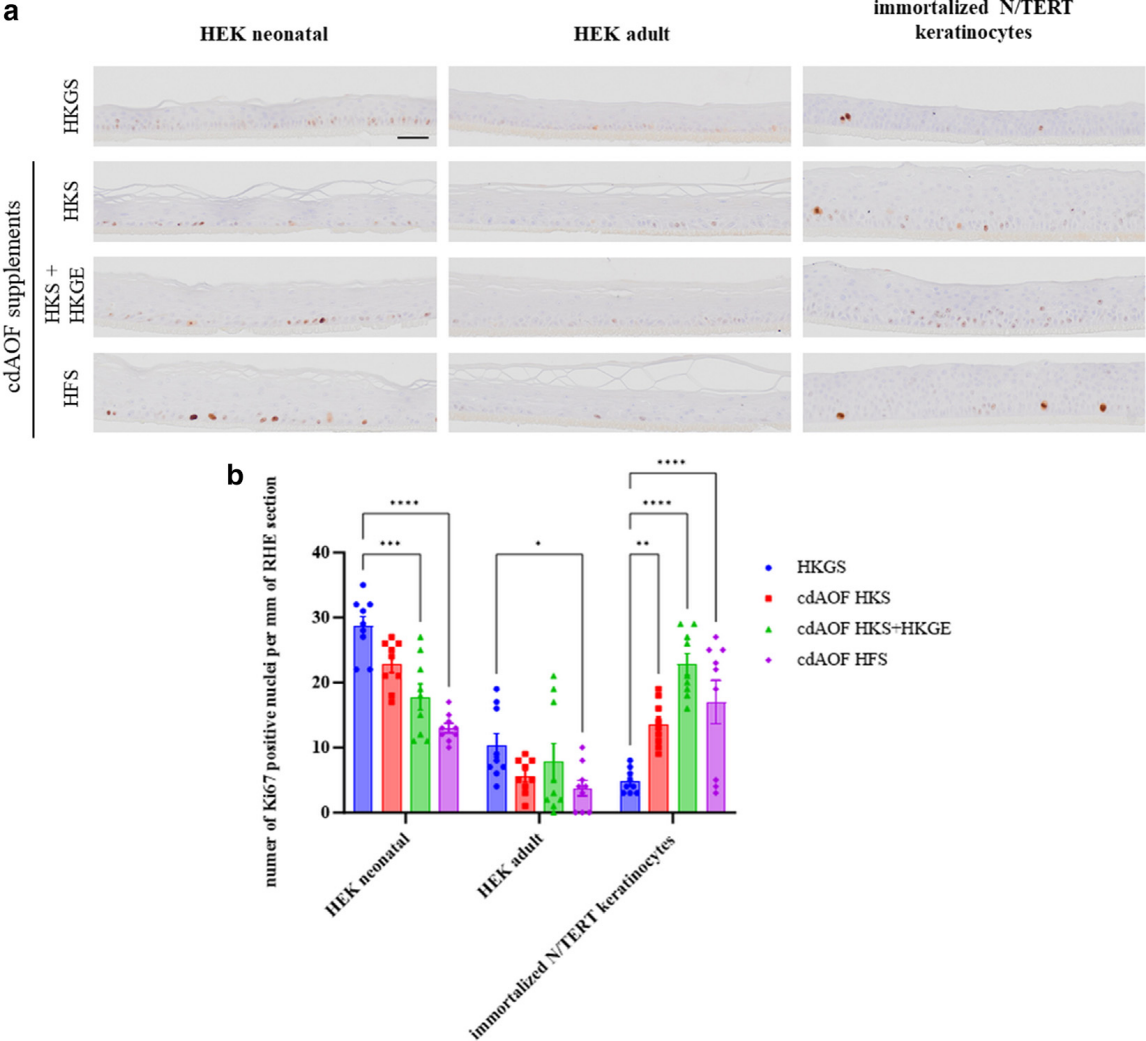
**Proliferation of keratinocytes in RHE via Ki67 immunolocalization.** (**a**) IHC staining for Ki67 revealed differences in proliferation marker intensity in skin models reconstructed with HEKn, HEKa, or N/TERT keratinocytes cultured with HKGS, HKS, HKS + HKGE, or HFS supplements (n = 3 independent experiments). Scale bar = 50 μm. Magnification (×100). (**b)** Quantification of proliferation marker Ki67 by double-blind test (n = 9). Quantification was made on three different areas of each skin model of every condition. Data represent mean ± SEM. Two-way ANOVA was performed, followed by Dunett`s tests. ∗*P* < .05, ∗∗*P* < .01, ∗∗∗*P* < .001, ∗∗∗∗*P* < .0001. HEK, human epidermal keratinocytes; HFS, human fibroblast supplement; HKGE, human keratinocyte growth supplement; HKGS, Human keratinocyte growth supplement; HKS; high keratinocyte serum-free medium; RHE, reconstructed human epidermis; TERT, telomerase reverse transcriptase.

The proliferative activity was evaluated using Ki67 marker-specific immunohistochemistry staining of the RHEs (Figure 2a). From the results, cell proliferation not only depends on the supplement used for epidermal reconstruction, but it also varies from one type of keratinocyte to another. Notably, keratinocytes obtained from a neonatal donor (HEKn) exhibited the highest frequency of Ki67 positive cells, along the basal layer, particularly when utilizing the HKGS supplement (Figure 2b). In contrast, N/TERT keratinocytes show the lowest proliferation rate when HKGS is used compared to primary keratinocytes. Additionally, HKS + HKGE and HFS supplements significantly contribute to reduced proliferation of keratinocytes in RHEs derived from HEKn. Moreover, the HFS supplement has a significant effect on the reduction of the proliferative capacity of HEKa keratinocytes when compared to HKGS. Interestingly, cdAOF supplements demonstrate an increased impact on the proliferation of N/TERT keratinocytes as compared to the animal-originated HKGS supplement.

### Differentiation marker of immunolocalization in RHEs derived from HEKn, HEKa or N/TERT keratinocytes

**Figure 3:**
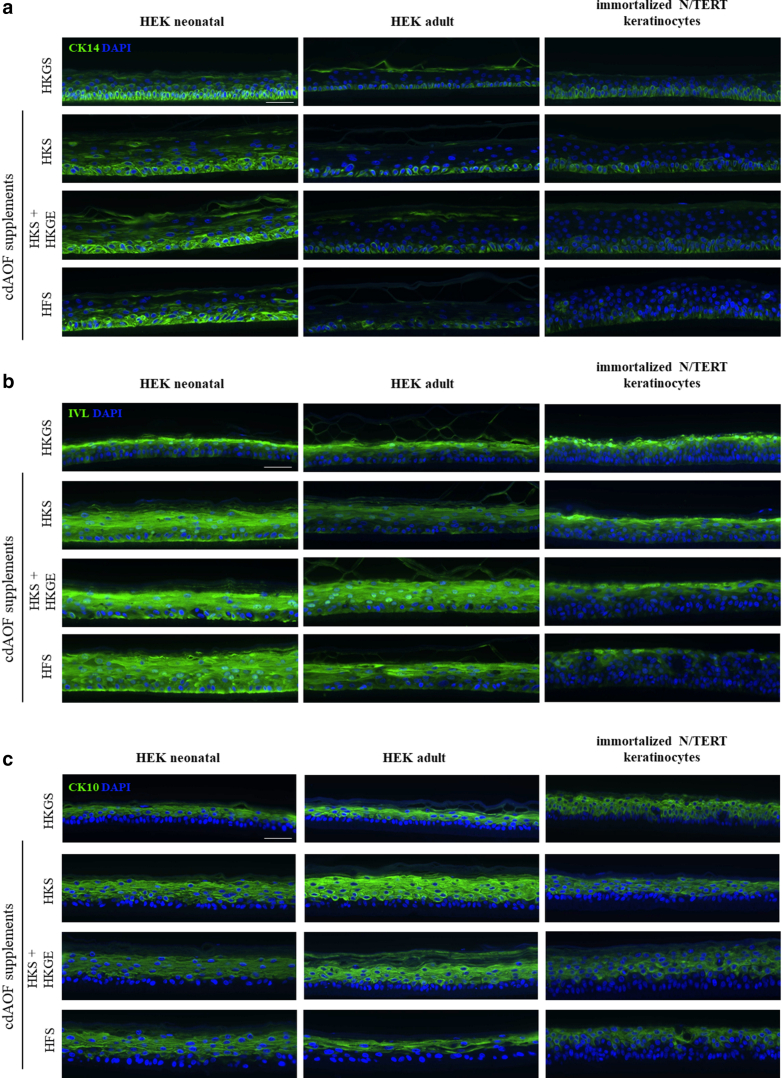

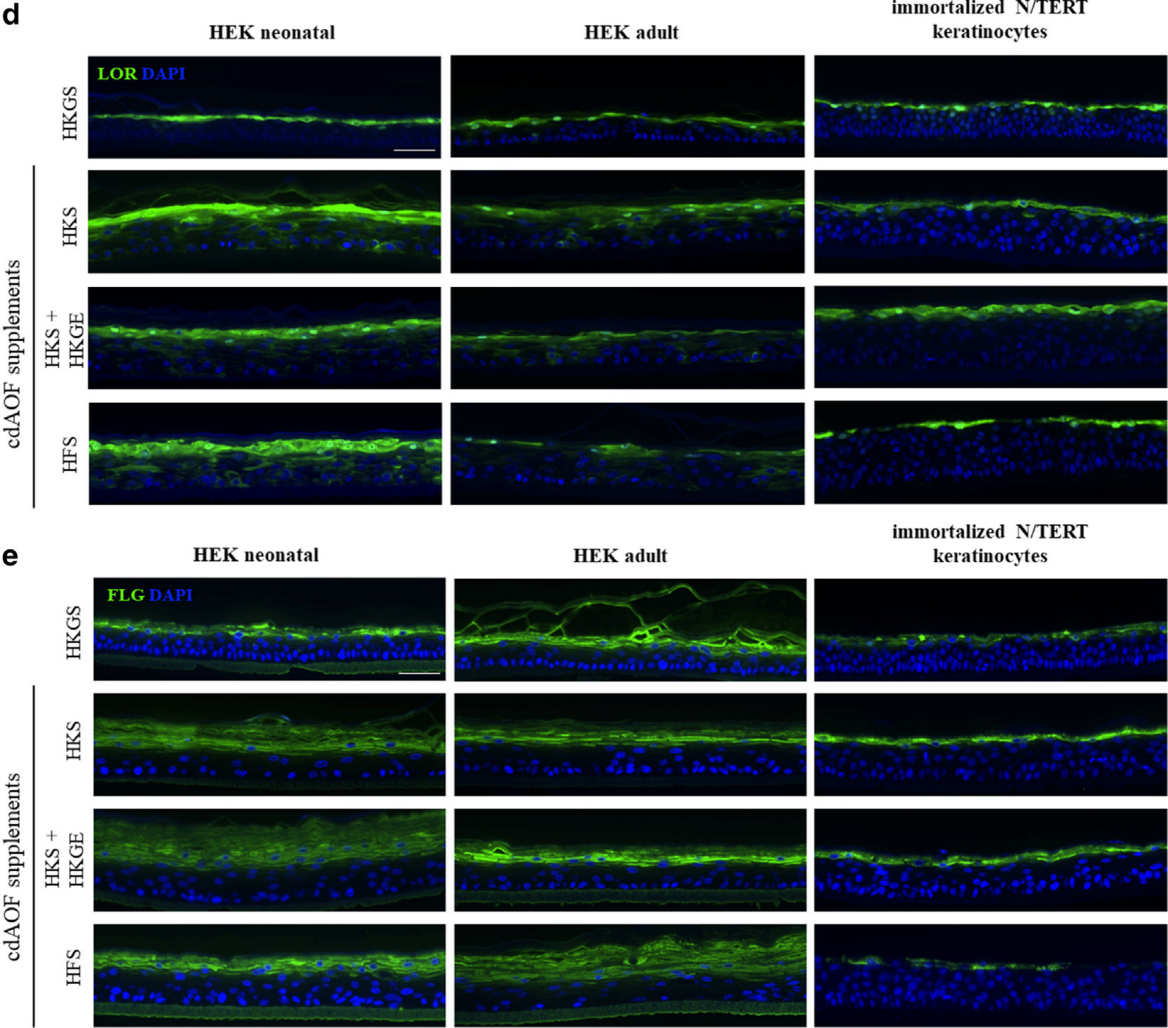
**Immunostaining and localization of differentiation-related markers in RHE.** (**a**) CK14. (**b**) IVL. (**c**) CK10 (**d**) LOR. (**e**) FLG. Nuclei were stained with DAPI (blue). Scale bar: 50 μm. Pictures are representatives of 3 independent experiments. CK, cytokeratin; IVL, involucrin; LOR, Loricrin; RHE, reconstructed human epidermis.

To assess differentiation-related endpoints after epidermal reconstruction, immunostaining of several differentiation markers was performed. Immunostaining indicated that RHE produced from HEKn more strongly expresses cytokeratin 14 (CK14), a marker of basal keratinocytes when compared to HEKa or N/TERT keratinocytes (Figure 3a). Interestingly, the expression of CK14 is not generally related to the type of cell culture supplement used for tissue reconstruction. When cultured with either HKGS or the cdAOF supplements, RHEs derived from HEKn display CK14 immunofluorescence that is generally present at a higher level, that clearly extends beyond the basal layer into the suprabasal layer, and to a lesser degree, into the stratum corneum. In contrast, CK14 immunofluorescence in RHEs constructed from HEKa is lower when using either HKGS or any of the cdAOF supplements, and displays CK14 expression in the basal layer and to a lesser degree within the stratum corneum. In contrast, in the case of N/TERT keratinocytes, CK14 appears to be more restricted to the basal layer when cultured with either HKGS or any of the cdAOF supplements. Immunolabeling of IVL indicated elevated and premature basal expression when HEKn-derived RHE was cultured with the cdAOF supplements HKS, HKS + HKGE, or HFS (Figure 3b). Additionally, this IVL-related phenomenon was most pronounced in RHE derived from HEKn, observed to a lesser degree in RHE derived from HEKa, and was not seen in N/TERT keratinocytes. Immunofluorescent staining of CK10 (Figure 3c), Loricrin (LOR) (Figure 3d), or FLG (Figure 3e) appear similar in RHE produced with cdAOF culture supplements and in RHE cultured with animal-originated products (HKGS), and in normal human skin as well (not shown).

### Elevated levels of mRNA for IL-1α and other markers indicate a partial inflammatory-proliferative gene expression profile in RHEs cultured under some cdAOF conditions

**Figure 4:**
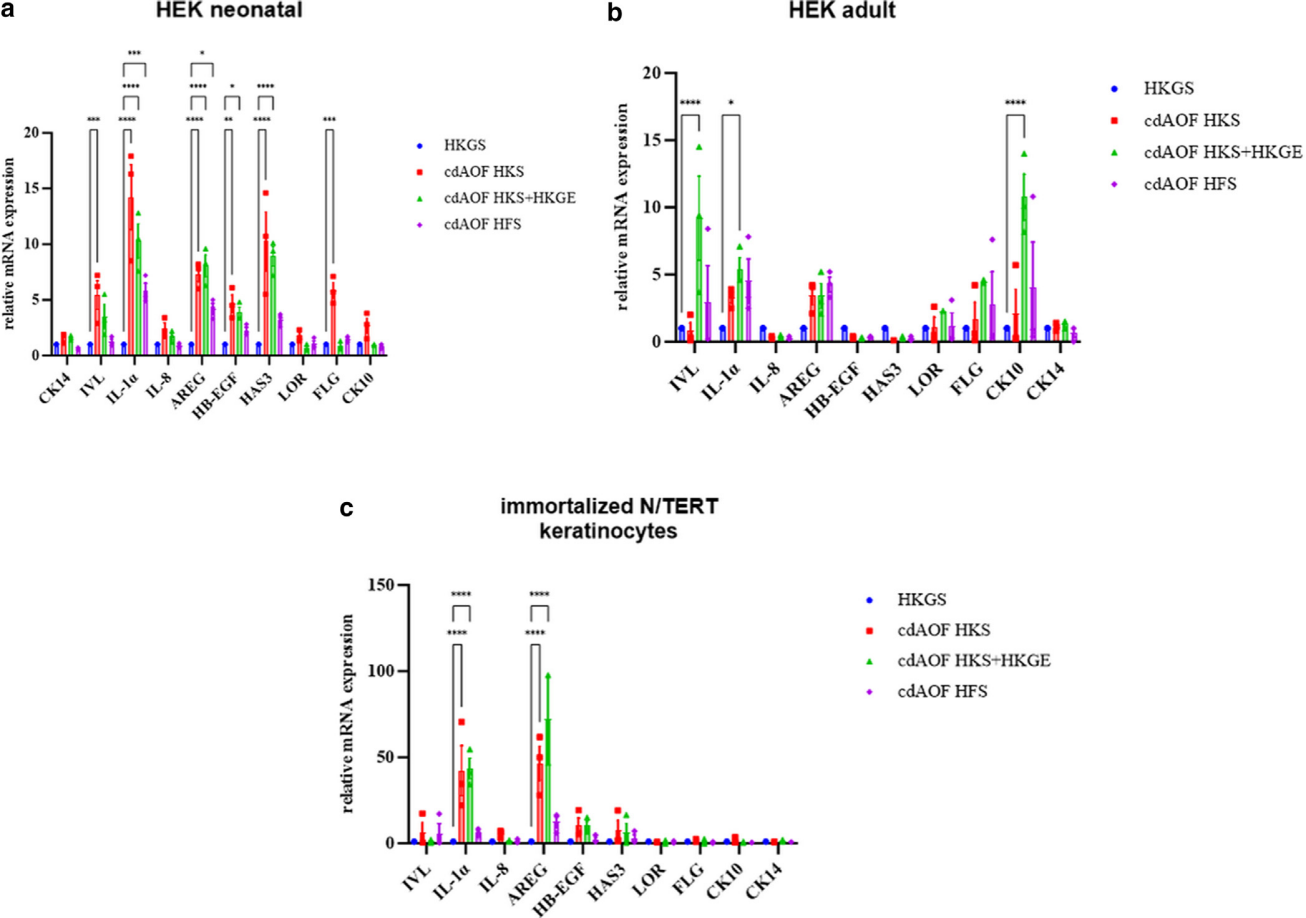
**Transcriptional study of genes related to epidermal differentiation, inflammation, and proliferation.** mRNA expression was assessed by RT-qPCR. Gene expression in RHE reconstructed of (**a**) HEKn, (**b**) HEKa, and (**c**) immortalized N/TERT keratinocytes. Data represent mean ± SEM (n = 3 independent experiments). A 2-way ANOVA test was performed, followed by Dunnett`s tests. ∗*P* < .05, ∗∗*P* < .01, ∗∗∗*P* < .001, ∗∗∗∗*P* < .0001. HEK, human epidermal keratinocytes; RHE, reconstructed human epidermis; TERT, telomerase reverse transcriptase.

As shown in Figure 4a, RHEs reconstructed from HEKn using the HKS supplements exhibited elevated levels of inflammatory-proliferative markers IL-1α (Kupper and Groves, 1995), amphiregulin (AREG), heparin-binding EGF-like growth factor (HB-EGF) and hyaluronan synthase 3 mRNA when compared to RHE cultured in HKGS. Similarly, RHE reconstructed from HEKn in the fibroblast-dedicated HFS supplement displayed elevated levels of IL-1α and AREG mRNA. The analysis of transcripts also demonstrated that hyaluronan synthase 3, AREG, and HB-EGF, 3 markers linked to inflammation, autocrine proliferation, inflammatory-proliferative skin pathologies such as psoriasis (Cook et al, 2004b; Macleod et al, 2021) or exposure of keratinocytes to Th2 cytokines (De Vuyst et al, 2018) were significantly increased in RHE produced with HEKn cultured in HKS or HKS + HKGE supplements. In contrast, differentiation markers in RHE derived from HEKn cultured in HKS displayed significant changes in FLG and IVL mRNA levels (Figure 4a). CK14, CK10, LOR, and IL-8 gene expression appeared to be similar in RHE derived from HEKn cultured in the cdAOF supplements and in RHE derived from HEKn cultured in HKGS.

RHE from HEKa cultured in HKS + HKGE also demonstrated increased levels of IL-1α mRNA (Figure 4b). Culturing RHE with the HKS + HKGE supplement was associated with a significant increase in the expression of CK10 and IVL transcripts (Figure 4b). RHE derived from N/TERT keratinocytes culture in HKS or HKS + HKGE produces a significantly elevated expression of IL-1α and AREG (Figure 4c). Finally, our data reveal no significant changes in the expression of IL-8, under any condition.

## Discussion

Reducing the direct use of animals as well as the use of cell culture products derived from animals or humans is an inevitable progression for conducting research in accordance with the 3Rs principle (Díaz et al, 2021). Additionally, this progression reduces the risk of adverse inflammatory reactions or infectious events in reconstructive and regenerative cutaneous therapies. Over time, reconstructed skin models have demonstrated significant improvement with respect to reproducibility and reliability. They are designed to mimic the structure and function of human skin, making them suitable substitutes for animal testing(Duval et al, 2003; Flaten et al, 2015; Kandárová et al, 2006). RHE is the simplest model dedicated to analyzing some epidermal properties in vitro (Poumay and Faway, 2023). However, the production of RHE models generally relies on the use of HKGS or other animal-originated (including human-originated) components. This raises concerns regarding not only animal cruelty for those cell culture systems using reagents such as bovine-derived BPE and fetal bovine serum but also safety (transmissible disease) concerns when animal and human components exposed to RHE tissue are used for reconstructive and regenerative medicine applications.

Additionally, considerable problems with the supply of various laboratory products, including HKGS supplements during the COVID-19 pandemic occurred, stagnating the research of many scientists in the RHE research field (Poumay and Faway, 2023). To test alternatives and obtain a wider perspective regarding the use of cdAOF supplements for RHE reconstruction, we tested three different types of human keratinocytes comparing HKGS against the commercially available cdAOF supplements. We note that RHE derived from N/TERT keratinocytes, or similarly immortalized keratinocyte cell lines, might reduce the cost of RHE production (Smits et al, 2017) and allow for easier genetic manipulation through techniques that include CRISPR-Cas DNA-editing (Evrard et al, 2021; Progneaux et al, 2023).

In the current study, we demonstrate that all the cdAOF supplements produce functional RHE from either HEKn, HEKa, or N/TERT keratinocytes. RHE produced with cdAOF supplements exhibits all the typical features of human skin morphology with respect to stratification of the basal, spinous, and granular layers, leading to a well-keratinized stratum corneum. Notably, the stratum corneum layer is thinner in RHEs produced from N/TERT keratinocytes but is nonetheless similar in thickness to what is observed in the epidermis reconstructed with N/TERT keratinocytes in HKGS.

We also note, that there are some differences when comparing RHE produced using cdAOF supplements to RHE produced using HKGS. Basal keratinocytes of the human epidermis normally exhibit a cylindrical shape (Yousef et al, 2023). However, in the current study, the animal-free cdAOF supplements produced RHEs displaying a less cylindrical basal layer, especially when HFS was used as an alternative to HKGS. However, we point out that less cylindrical basilar keratinocytes have been observed in other, frequently utilized, well-known RHE models as well (Costin et al, 2009; MatTek Corp. EpiDerm: https://www.mattek.com/mattekproduct/epiderm/). Additionally, HFS still promoted epidermal reconstruction, even though the HFS formulation was developed specifically for dermal fibroblast culture, rather than for epidermal keratinocyte culture. IVL is a late differentiation marker in human skin and normally localizes in the upper spinous and granular layers (Furue, 2020). Using immunofluorescence to study IVL localization in RHE, expression was found in the basal layer of RHE cultured using cdAOF supplements, much earlier than normally observed in human skin and other RHE models (Figure 3b). Premature, enhanced expression of IVL occurs in some skin disorders linked to an imbalance in the keratinization process (Kanitakis et al, 1987). For instance, a similar premature distribution of IVL was found in RHE derived from the keratinocytes of a patient suffering from Darier disease (Lambert de Rouvroit et al, 2013). Additionally, some abnormalities were found in the immunoreactivity of CK14, which is a marker of proliferative keratinocytes in the basal layer (Vaidya and Kanojia, 2007). CK14 was detected predominantly in the basilar layer, in RHEs derived from HEKn when cultured with either animal component-derived (HKGS) supplement or the cdAOF supplements (Figure 3a). Furthermore, we also observed that CK14 in HEKa-derived RHEs was also detected suprabasally, in the stratum corneum of all the HEKn-derived RHE. With weaker immunofluorescent signals, similar patterns of CK14 were detected in HEKa-derived RHE as well. CK10, a marker of early differentiation in the suprabasal layers, appears correctly localized in RHE cultured with cdAOF supplements (Figure 3c). Similarly, other markers of late epidermal differentiation, such as LOR and FLG, are normally expressed in the granular layer of cdAOF RHEs (Figure 3d and e), bringing us to conclude that all the cdAOF supplements conferred production of a structurally functional RHE. This is also supported by the fact that HKS + HKGE was utilized as a cdAOF epidermal cell bioink to generate a xenofree, fully vascularized, full-thickness 3-Dimensional-bioprinted skin equivalents successfully engrafted onto mice (Baltazar et al, 2023).

Using the Ki67 marker, our current study further revealed that RHE derived from N/TERT keratinocytes (Figure 2b) exhibited increased proliferation rates when using cdAOF supplements as compared to the animal product-containing HKGS supplement. Using Ki67 immunolocalization, primary keratinocytes in RHE derived from HEKn and HEKa show greater proliferation rates when cultured with HKGS containing animal-originated products (BPE), than with cdAOF supplements. Interestingly, our study also suggests that the frequency of Ki67-positive cells is closely related to the type of keratinocytes used to produce the RHE. Still, TEER analysis revealed that barrier formation is generally more complete when HKGS is used to generate an RHE, as compared to the use of the cdAOF supplements. RT-qPCR results (Figure 4) reveal that some of the models reconstructed with cdAOF supplements exhibit signs of an inflammatory-proliferative response since increased levels of IL-1α, HA3, HB-EGF, and AREG mRNA are present.

In summary, within the limits of this study, when cultured with the cdAOF supplements HKS (HKSdaFREE) or HKS + HKGE, RHEs derived from HEKn and HEKa reproduce many, but not all the structural and functional properties of RHE reared in the HKGS environment. These similarities include a histological microanatomy displaying a multilevel stratified epidermis with differential expression and immunolocalization of proteins associated with epidermal differentiation and stratification (e.g. CK14, CK10, LOR, IVL, and FLG). TEER values are relative indicators of barrier function in RHE. In the current study, similarities also included statistically similar TEER values from HEKn-derived RHEs cultured in HKS without HKGE. In contrast to HEKn-derived RHEs cultured in HKS without HKGE, significantly lower TEER values were observed in both HEKn-derived RHEs cultured in HKS with HKGE and HEKa-derived RHEs cultured in HKS with or without HKGE. Still, other differences included elevated and premature IVL expression in both HEKn- and HEKa-derived RHEs cultured in HKS, with or without HKGE. Notably, as seen in commercially available RHE models, fewer columnar basal keratinocytes were observed in HEKn- and HEKa-derived RHEs cultured in HKS, with or without HKGE as well. Elevated transcripts for inflammatory-proliferative markers were also observed, as increased expression of IL-1α, hyaluronan synthase 3, AREG, and HB-EGF were noted in HEKn-derived RHEs cultured in HKS, whereas HEKa-derived RHEs demonstrated only increased expression of IL-1α mRNA when cultured in HKS without HKGE. Since the cdAOF supplements HKS and HKGE were experimentally developed to promote the rapid and efficient primary isolation and serial expansion of human skin-derived epidermal keratinocytes for regenerative medicine applications (Baltazar et al, 2023), it may not be surprising that inflammatory-proliferative transcripts are elevated in RHEs that are cultured in HKS. Identification of the specific proprietary components included in the HKS + HKGE formulation that might be responsible for the enhanced inflammatory-proliferative transcript profile, as well as the premature overexpression of IVL and lower TEER values, are currently under investigation. Indeed, preliminary unpublished evidence suggests that removal or reduction of specific HKS and HKGE components may favorably diminish both the elevated and displaced IVL expression and the high inflammatory-proliferative transcript expression as well.

The use of cdAOF cell culture supplements for epidermal reconstruction marks significant progress in the field of animal cruelty-free cosmetics, cosmeceuticals, aesthetic medicine, and safer regenerative medicine as well. This highlights the need for more advanced animal-origin-free biomedical research and development within these areas. In addition, animal products are prone to lot-to-lot variation, which can often be a source of uncertainty in the method and must be extensively tested before being introduced into routine procedures. In cdAOF products such lot-to-lot variation is greatly reduced, which allows for better standardization of the process. At the same time, there is an important economic aspect, as animal-free products reduce the time and cost of finding a well-suited lot. Moreover, the availability of cdAOF cell culture systems provides alternatives to the supply chain interruption of conventional animal product-containing cell culture supplements.

Finally, the cdAOF approach not only aligns with ethical considerations to reduce the general use of animal-originated products but also offers other compelling advantages, such as reducing the risk of transmission of both human-originated and zoonotic diseases during regenerative medical procedures.

## Materials and Methods

### Origin of cells

HEKa were isolated from abdominoplasty (Clinique St Luc, Namur, Belgium), as described (Minner et al, 2010). Samples from patients were obtained after written informed consent and in agreement with the principles and guidelines of the Declaration of Helsinki and approved by the Ethics committee of the Clinique Saint-Luc, Bouge (Namur). HEKn was purchased from ThermoFisher Scientific (Cat#C0015C, Massachusetts). Immortalized N/TERT-G2 keratinocytes were obtained from the J. Rheinwald laboratory (Harvard Medical School, Boston, MA) (Dickson et al, 2000).

### Culture condition and skin reconstruction

HEKa, HEKn and N/TERT keratinocytes were routinely cultured in EpiLife medium (ThermoFisher Scientific Cat#MEPI500CA) supplemented with HKGS (ThermoFisher Scientific Cat#S0015), penicillin 50 U/ml and streptomycin 50 μg/ml (Millipore Sigma, Cat#P4333, Missouri, MI) until reaching 70-80% of confluency. Keratinocytes were harvested, then, for epidermal reconstruction, seeded at a density of 330,000 cells/cm^2^ onto a polycarbonate filter with a pore size of 0.4 μm, as previously described (Poumay et al, 2004). To reconstruct the epidermis, the EpiLife medium with 4 different supplement formulations (see Table 1) were used as follows: (i) HKGS containing 0.2 % v/v BPE, 0.01 μg/ml, recombinant human insulin-like growth factor-I, 0.18 μg/mL hydrocortisone, 5 μg/ml bovine transferrin, 0.2 ng/ml human epidermal growth factor; (ii) HKSdaFREE (HKS), specific for human keratinocytes and corneal epithelial cells (AvantBio Inc, Cat#AVB01HKS, Lynnwood); (iii) HKSdaFREE + HKGE (HKS + HKGE) (AvantBio Inc, Cat#AVB01HKS) and (iv) HFSdaFREE (HFS), specific for human fibroblasts (AvantBio Inc, Cat#AVB02HFS). All media used for the culture of RHEs received an additional 1.44 mM CaCl₂ to reach 1.5 mM Ca^2^⁺ final concentration. After 24 hours of incubation at 37 °C in a humidified atmosphere containing 5% carbon dioxide, seeded cultures were exposed to the air-liquid interface. Media were additionally supplemented with 50 μg/ml vitamin C (Sigma-Aldrich Cat#49752, Saint Louis) to sustain the formation of stratum corneum barrier lipids (Ponec et al, 1997) and 10 ng/ml keratinocyte growth factor (R&D Systems Cat#251KG, Minneapolis). RHEs were cultured for 10 additional days with media being changed every 2 to 3 days.

### H&E staining

After 11 days of reconstruction, histological preparation of RHE and H&E staining of tissue sections were performed as previously described (De Vuyst et al, 2013). Briefly, the RHEs were fixed in 4% acetic formalin, dehydrated, and embedded in paraffin. 6 μm thick tissue sections were cut and placed onto microscope slides. After deparaffinization and rehydration, sections of RHE were stained with H&E, dehydrated, and mounted with DPX (VWR International, Leuven, Belgium).

### Immunohistological Staining

For immunolabeling, RHE sections were deparaffinized, rehydrated, and rinsed with tap water. To detect specific proteins, the following primary antibodies were used: CK14 (mouse, monoclonal, IgG, dilution 1:50; Santa Cruz Cat#sc58724), CK10 (mouse, monoclonal, IgG1, dilution 1:100; Dako Cat#M7002, Glostrup, Denmark), IVL (mouse, monoclonal, IgG1, dilution 1:200; Millipore Sigma Cat#I9018, MO), LOR (rabbit, polyclonal, IgG, dilution 1:100; Abcam Cat#ab24722, Cambridge, United Kingdom), filaggrin (mouse, monoclonal, IgG, dilution 1:75; ThermoFisher Scientific Cat#MA5-13440, Waltham) and Ki67 (mouse, monoclonal, IgG1, dilution 1:50; Diagomics Cat#M3034, Blagnac Cedex, France). For immunofluorescence the secondary antibody used was Alexa 488-conjugated anti-mouse (goat, polyclonal, IgG, dilution 1:200; Invitrogen Cat#A11001, Carlsbad) or Alexa 488-conjugated anti-rabbit (goat, polyclonal, IgG, dilution 1:200; Invitrogen Cat#A11008, Carlsbad). For immunohistochemistry, the anti-rabbit Vectastain ABC HRP kit (goat, polyclonal, IgG, dilution 1/200; Vector Laboratories Cat#PK4001) was used.

### TEER measurement

On the 11th day of RHE culture, TEER was measured using the Millicell ERS-2 device (Millipore Corporation, Burlington, MA).

### RNA extraction and RT-qPCR

RNA isolation and RT-qPCR were performed as previously described (Progneaux et al, 2023). Briefly, RNA from RHE was extracted using the “ReliaPrep miRNA Cell and Tissue Miniprep System” (Promega Cat#Z6111), and its concentration was measured by NanoDrop 1000 spectrophotometer (ThermoFisher Scientific). cDNA was then synthesized using the “SuperScript III RNase H-Reverse Transcriptase Kit” (Thermo Fisher Scientific Cat#18080093, Massachusetts). SYBR Green method (Eurogentec, Liège, Belgium) and specifically designed primers (Eurogentec, Liège, Belgium; Millipore Sigma) listed in Table 2 were used. Gene expression is normalized to the expression of ribosomal protein P0 as a reference (housekeeping) gene (Minner et al, 2010) to control the variability in expression levels and was analyzed using the 2^–^ΔΔCT method (Livak and Schmittgen, 2001). Fluorescence of SYBRgreen was measured using LightCycler 96 (Roche Diagnosis, Vilvoorde, Belgium).

**Table 2:** Sequences of Primers Used for RT-qPCR

Interest Gene	Forward	Reverse	Company
RPLP0	ATCAACGGGTACAAACGAGTC	CAGATGGATCAGCCAAGAAGG	Eurogentec, Liège, Belgium
CK14	CGATGGCAAGGTGGTGTC	GGGTGAAGCAGGGTCCAG	Eurogentec, Liège, Belgium
CK10	AATCAGATTCTCAACCTAACAAC	CTCATCCAGCACCCTACG	Sigma-Aldrich, MO, USA
IVL	TGAAACAGCCAACTCCAC	TTCCTCTTGCTTTGATGGG	Eurogentec, Liège, Belgium
FLG	GGGCACTGAAAGGCAAAAAG	CACCATAATCATAATCTGCACTACCA	Eurogentec, Liège, Belgium
LOR	TCATGATGCTACCCGAGGTTTG	CAGAACTAGATGCAGCCGGAG	Eurogentec, Liège, Belgium
IL-1α	AACCAGTGCTGCTGAAGGAGAT	TGGTCTCACTACCTGTGATGGTTT	Eurogentec, Liège, Belgium
IL-8	TTGGATACCACAGAGAATGAA	GCAGAGGGTTGTGGAGAAGTT	Eurogentec, Liège, Belgium
AREG	ATAGAGCACCTGGAAGCAGTAACA	ACTTTTCCCCACACCGTTCA	Sigma-Aldrich, MO, USA
HBEGF	TGGCCCTCCACTCCTCATC	GGGTCACAGAACCATCCTAGC	Sigma-Aldrich, MO, USA
HAS3	GCGATTCGGTGGACTACAT	GGATCCTCCTCCAGGACTC	Sigma-Aldrich, MO, USA

Abbreviations: AREG, amphiregulin; CK, cytokeratin; HAS3, hyaluronan synthase 3; IVL, involucrin; LOR, Loricrin; RPLP0, ribosomal protein P0.

### Statistical analysis

Statistical differences were determined using a two-way ANOVA test followed by Dunett´s post hoc test and carried out using GraphPad software (v 10.1.2, GraphPad Software, Inc). A *P* value of < .05 was considered significant.

## Ethics Statement

The collection of primary adult keratinocytes from human skin (HEKa) was approved by the Ethics committee of the Clinique Saint-Luc, Bouge (Namur).

## Data Availability Statement

All data can be available upon request to yves.poumay@unamur.be.

## ORCIDs

Julia Bajsert: http://orcid.org/0009-0002-9094-4592

Valérie De Glas: http://orcid.org/0000-0001-7366-2601

Emilie Faway: http://orcid.org/0000-0003-3406-1103

Catherine Lambert de Rouvroit: http://orcid.org/0000-0002-0273-8995

Miguel Pérez-Aso: http://orcid.org/0000-0002-4703-4182

Paul W. Cook: http://orcid.org/0009-0001-7079-4753

Yves Poumay: http://orcid.org/0000-0001-5200-3367

## Conflict of Interest

PWC is the founder and owner of AvantBio. The remaining authors state no conflict of interest.
